# How the cerebellum may monitor sensory information for spatial representation

**DOI:** 10.3389/fnsys.2014.00205

**Published:** 2014-11-04

**Authors:** Laure Rondi-Reig, Anne-Lise Paradis, Julie M. Lefort, Benedicte M. Babayan, Christine Tobin

**Affiliations:** ^1^Sorbonne Universités, UPMC Univ Paris 06, UMR-S 8246/UM 119, Neuroscience Paris Seine, Cerebellum, Navigation and Memory TeamParis, France; ^2^Institut National de la Santé et de la Recherche Médicale 1130, Neuroscience Paris Seine, Cerebellum, Navigation and Memory TeamParis, France; ^3^Centre National de la Recherche Scientifique, UMR 8246, Neuroscience Paris Seine, Cerebellum, Navigation and Memory TeamParis, France

**Keywords:** cerebellum, self-motion, navigation, place cells, head direction cells, parietal cortex, sensory processing, hippocampus

## Abstract

The cerebellum has already been shown to participate in the navigation function. We propose here that this structure is involved in maintaining a sense of direction and location during self-motion by monitoring sensory information and interacting with navigation circuits to update the mental representation of space. To better understand the processing performed by the cerebellum in the navigation function, we have reviewed: the anatomical pathways that convey self-motion information to the cerebellum; the computational algorithm(s) thought to be performed by the cerebellum from these multi-source inputs; the cerebellar outputs directed toward navigation circuits and the influence of self-motion information on space-modulated cells receiving cerebellar outputs. This review highlights that the cerebellum is adequately wired to combine the diversity of sensory signals to be monitored during self-motion and fuel the navigation circuits. The direct anatomical projections of the cerebellum toward the head-direction cell system and the parietal cortex make those structures possible relays of the cerebellum influence on the hippocampal spatial map. We describe computational models of the cerebellar function showing that the cerebellum can filter out the components of the sensory signals that are predictable, and provides a novelty output. We finally speculate that this novelty output is taken into account by the navigation structures, which implement an update over time of position and stabilize perception during navigation.

## Introduction

The ability to maintain a sense of direction and location while moving in one's environment is a fundamental cognitive function. Humans and more generally animals rely on a spatial cognitive process in complex environments for obtaining food, avoiding dangers and finding their nest/home. The cerebellum has been shown to participate in this spatial cognitive process (see review in Petrosini et al., 1998; Schmahmann and Sherman, 1998; Rondi-Reig et al., 2002; Rondi-Reig and Burguière, 2005). However, the computational processes supported by the cerebellum in that function and its anatomo-functional links with more traditional navigation structures are still debated.

Neuronal navigation circuits have been described in various behaviors ranging from exploration to goal-directed navigation. Those circuits underlie the acquisition of knowledge about the environment through different elementary processes we can exemplify by imagining the following situation. When one arrives in a new city, one may wander around, gathering and memorizing information about salient and/or recognizable landmarks, either proximal (this red house, the hairdresser…) or visible from a distance (distal; a tower, a church, a hill…). One can then use this information to get to a place by either moving toward a distantly visible monument or by trying to remember the succession of direction changes performed from the departure point, possibly at the recognizable landmarks. When the city becomes well-known, other elementary processes may take place and allow the navigator to use the knowledge previously acquired. Indeed, Spiers and Maguire (2006) have shown that after initially planning the route to our destination, we set up expectations, waiting to see a particular landmark to check if we are on the right route, we occasionally inspect the city around us as we travel through it (“this building has been cleaned”), and we may also see an opportunity to adjust our route if necessary. If driving, we also continuously monitor the surrounding traffic to achieve safe passage to our destination and plan actions, such as changing lanes.

This detailed description reveals the complexity of navigation and the multiplicity of sub-processes that can vary in time depending on the amount of knowledge one has of one's environment and the given navigational constraints.

Interestingly, all these sub-processes of navigation rely on sensory processing to provide the navigator with information about their position and orientation in the environment. Much of the information about where we are is known to come from external or allothetic cues. However, navigation also generates self-motion—also called idiothetic—information when one is moving in this environment. Monitoring such self-motion information is essential to estimate one's movement in space to update one's position and orientation.

The continuous monitoring of this self-motion information may subserve the mental handling of spatial knowledge. This review will focus on the engagement of the cerebellum in this continuous process.

To this end, we will first review the sensory inputs to the cerebellum and the cerebellar outputs directed toward navigation circuits. We will then discuss the computational algorithm(s) thought to be performed by the cerebellum in this context. Finally, we will review how self-motion signals influence information coded in these navigation circuits. Recent literature now provides anatomo-functional descriptions of the cerebellum at the micro-circuit level, revealing microcomplexes depending on the zebrin-histochemical status of the Purkinje cells, longitudinal zones and microzones of the cerebellar cortex (Hawkes and Herrup, 1995; Pijpers et al., 2006; Cerminara et al., 2013) and subdivision of the inferior olivary and deep cerebellar nuclei (Garwicz et al., 1998; Sugihara and Shinoda, 2004; Pijpers et al., 2005, see also Apps and Watson, 2013 for a review). However, the present literature describing cerebellar links with forebrain areas is much less detailed. The functional links proposed in this review will therefore be described at a macroscopic level.

## Monitoring self-motion information for navigation

The importance of monitoring self-motion information during navigation was first revealed by Mittelstaedt and Mittelstaedt (1980) who studied the ability to navigate without external cues. They tested the ability of gerbils to retrieve their pups from within a circular arena and then return to their nest at the arena border. After using sometimes convoluted paths to initially find their pups, the gerbils then returned to their nests using direct paths, even in darkness. This behavior suggested that gerbils could integrate their movements to calculate a direct vector toward their departure. When the gerbils were slowly rotated on a platform (with an angular acceleration below the vestibular threshold, and hence not detected by the animals) while picking up a pup, they returned “home” in a direction that deviated from the nest by the amount they had been rotated. In other words, they homed using an internal (and in this case disrupted) sense of direction rather than external references. This ability was called path integration (Mittelstaedt and Mittelstaedt, 1980).

It is noteworthy that animals can rely on self-motion even when external information is available. This was highlighted by studies showing that an animal can find its goal in the darkness after learning it with a landmark or inducing a conflict between external and self-motion cues (Etienne and Jeffery, 2004; Rochefort et al., 2011). Therefore, self-motion information appears to be constantly available and effectively used whatever the navigation constraints.

Self-motion cues are provided by several systems: vestibular (translational and rotational accelerations) (Stackman and Herbert, 2002; Zheng et al., 2007), proprioceptive (feedback information from muscles, tendons, and joints), visual in the presence of light (linear and radial optic flow) (Etienne and Jeffery, 2004), acoustic (Valjamae, 2009) and even tactile (tactile flow) (Bremmer, 2011; Schroeder and Hartmann, 2012). It has also been suggested that during an active movement, while the motor cortex sends a motor command to the periphery, a copy of this command (called an efference copy) is also generated and sent to the cerebellum where it could be used to generate a prediction of the sensory consequences of the intended movement (Holst and Mittelstaedt, 1950).

In the following section we propose a schematic description of the anatomical pathways that convey self-motion information to the cerebellum. We will specify the intermediate relay nuclei from sensors to cerebellum and the main lobules receiving this multi-source information. The anatomical description is restricted to rodents and rabbits, with inputs from primates which provide extensive electrophysiological functional data.

## Sensory inputs to the cerebellum

The principal inputs to the cerebellar cortex are mossy fibers and climbing fibers. Mossy fibers originate from the spinal cord and from a wide range of nuclei in the brain stem, namely the pontine, vestibular, trigeminal and dorsal column nuclei. These mossy fibers convey information to the cerebellum from peripheral sensors located on body and head, and from cerebral cortices (see for review Ruigrok, 2004).

Sensory information entering the cerebellar cortex via mossy fibers is then distributed to, and integrated by, granule cells in the granular layer, which in turn excite the principle output of the cerebellar cortex, Purkinje cells, as well as interneurons within the molecular layer (stellate cells and basket cells).

The climbing fibers constitute the other main afferent to the cerebellar cortex. They arise exclusively from the inferior olive, a well defined nucleus in the ventral part of the brainstem. The axon of an olivary neuron divides into several branches that terminate in the molecular layer where they wrap around the dendritic tree of a Purkinje cell and make numerous synaptic contacts. Remarkably, in adults rats, each Purkinje cell is contacted by only one climbing fiber but each climbing fiber contacts seven Purkinje cell on average (Armstrong and Schild, 1970). The inferior olive receives information from many sources, including the dorsal column nuclei, the prepositus hypoglossi nucleus (PrH) (McCrea and Horn, 2006), the spinal trigeminal nuclei (Van Ham and Yeo, 1992; Yatim et al., 1996), the superior colliculus (May, 2006) and the cerebral cortex, mainly the sensori-motor cortex (Baker et al., 2001; Azizi, 2007; Watson et al., 2013).

**Figure 1:**
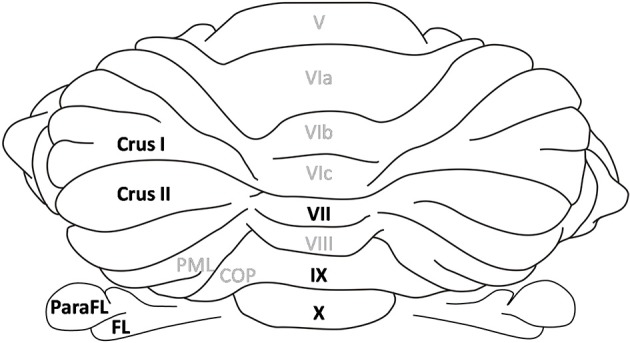
**Cerebellar lobules**. Dorsal view of the rat cerebellum. Lobules in the vermis are numbered according to Larsell's schema (Larsell, 1952). The lobules discussed in the review are highlighted in black. The flocculus and paraflocculus correspond to the hemispheric part of the flocculo-nodular lobe whereas lobules IX and X refer to its vermal part. Similarly Crus I and II are hemispheric regions in the posterior lobe whereas lobule VII is the corresponding vermal lobule. ParaFL, paraflocculus; FL, flocculus; PML, paramedian lobe; COP, copula pyramidis.

**Figure 2:**
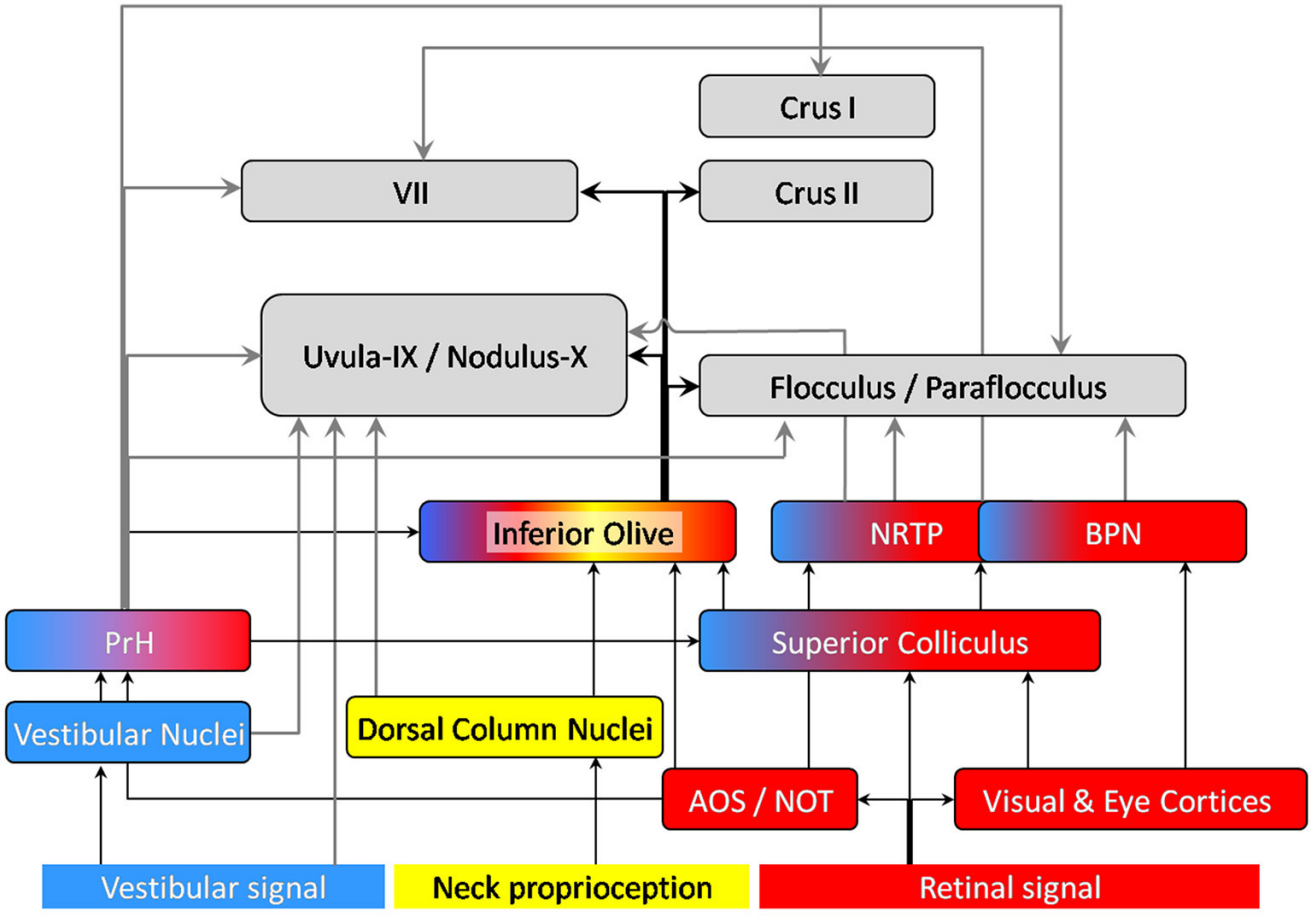
**Anatomical projections of visual (red), vestibular (blue) and neck proprioception (yellow) inputs to the cerebellar cortex**. The displayed connections were found in rodents and/or rabbits. Arrows connecting the cerebellum are highlighted in bold: gray arrows correspond to mossy fibers, black ones to climbing fibers. AOS, Accessory Optic System; NOT, Nucleus of the Optic Tract; BPN, Basilar Pontine Nuclei; NRTP, Nucleus Reticularis Tegmenti pontis; PrH, Prepositus Hypoglossi Nucleus. Note that the vermal regions of the cerebellar cortex (lobule VII, Uvula-Nodulus) are represented on the left whereas the hemispheric regions are represented on the right (Crus I, II, flocculus-paraflocculus).

**Figure 3:**
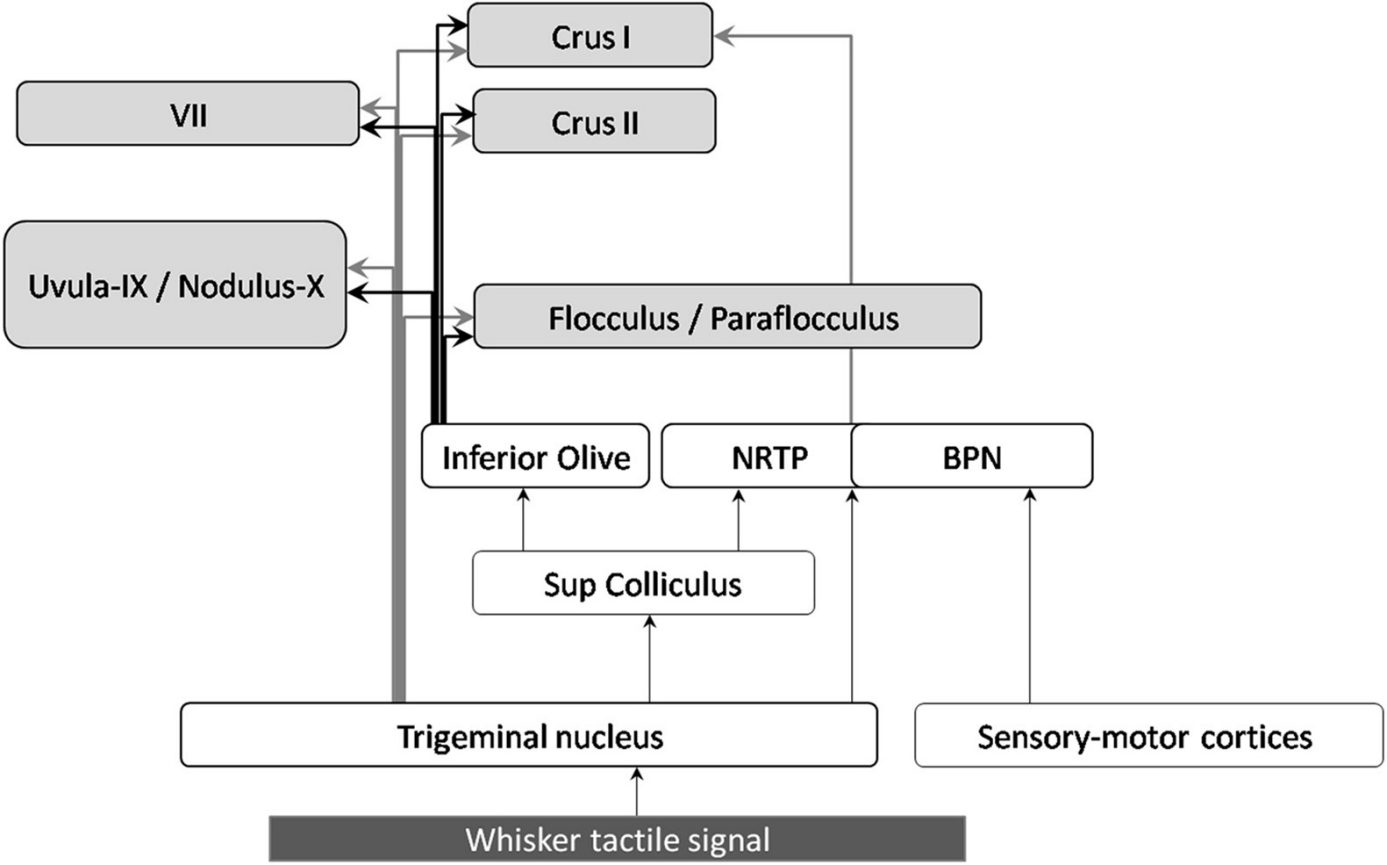
**Anatomical projections of whisker tactile inputs to the cerebellar cortex**. The displayed connections were found in rodents and/or rabbits. Arrows connecting the cerebellum are highlighted in bold: gray arrows correspond to mossy fibers, black ones to climbing fibers.

In the following we briefly detail anatomical projections of visuo-vestibular and neck proprioception signals which are well documented in terms of pathway and computational combination in optokinetic and vestibulo-ocular reflexes. We also describe whisker signal afferences, the processing of which is usually considered independently from that of visual and vestibular signals. We will question whether a convergence may exist between those signals within the context of navigation, and whether the efference copy signal, which targets the cerebellum and circuits engaged in self motion information process, may also be integrated with sensory signals in the cerebellum. The cerebro-cerebellar pathway which transmits already processed information, in particular from the sensory and associative cerebral cortices (Morissette and Bower, 1996) will not be described here. We will focus our description on the inputs to the vestibulocerebellum lobules IX, X (Figure 1), to the flocculus and paraflocculus, as well as the posterior lobules (VII, Crus I, Crus II) of the cerebellar cortex, which are often associated with the involvement of the cerebellum in cognitive functions (see Buckner, 2013 for a review). Figures 2, 3 illustrate the anatomical projections of these sensory inputs to the cerebellar cortex in rodents and rabbits.

### Vestibular projections

Vestibular inputs to the cerebellar cortex have two main origins. A small projection from the vestibular sensors, called the primary afferents, directly reach the ipsilateral uvula-nodulus. A larger projection, called the secondary afferents, pass through the vestibular nuclei before reaching different cerebellar lobules.

In rabbits, most of the mossy fibers directly originating from the vestibular sensors reach the uvula-nodulus (Barmack et al., 1993). The uvula-nodulus also receives indirect vestibular signals though the prepositus hypoglossi nucleus (PrH), which also sends ascending projection to the flocculus (Thunnissen et al., 1989).

In rodents, vestibular signals relayed by various vestibular nuclei not only project to the uvula-nodulus but also project to the flocculus, paraflocculus and lobule VII via the PrH (Päällysaho et al., 1991; Ruigrok, 2003).

Vestibular climbing fibers originate from two subnuclei of the inferior olive, the β-nucleus and the dorsomedial cell column. The outputs of these olivary nuclei terminate in the contralateral uvula-nodulus (See review in Barmack, 2003) and in the flocculus (Schonewille et al., 2006). The inferior olive receives vestibular inputs from the vestibular nuclei as well as the PrH (Gerrits et al., 1985). Indeed, the PrH nucleus does not belong to the vestibular complex, it does however receive numerous vestibular afferents from the vestibular nuclei (Baker and Berthoz, 1975; McCrea and Horn, 2006).

### Visual projections

Visual inputs are received by two areas of the posterior cerebellar cortex, lobule VII and the dorsal paraflocculus (see review Kralj-Hans et al., 2007).

In monkey, the basilar pontine nuclei (BPN), which receive inputs from cortices involved in eye movements (e.g., the frontal eye fields) and the perception of visual motion, sends mossy fiber projections to the dorsal paraflocculus (Giolli et al., 2001) and to lobule VII. Some projections to lobule VII also come from the nucleus reticularis tegmenti pontis (NRTP). Both the basilar pontine nuclei and the NRTP receive visual and oculomotor inputs from the cerebral cortex via the superior colliculus (see review Voogd and Barmack, 2006).

More visual information also reaches the cerebellum through mossy fibers from PrH. This structure, known to be involved in eye velocity and gaze signals, receives ascending projections from the Accessory Optic System (AOS). AOS is known to receive retinal signals related to the speed and direction of movement of large, textured visual patterns (AOS) (Soodak and Simpson, 1988) and is proposed to be dedicated to the processing of “optic flow fields” (Wylie et al., 1999). It has been shown to detect self-motion rather than the motion of external objects (Simpson et al., 1988). In sum, mossy fibers conveying visual information mainly arise from the BPN, the NRTP, and the PrH.

*Climbing fibers* projecting to the dorsal paraflocculus and lobule VII originate from different sub-parts of the inferior olive. Major projections to the dorsal paraflocculus arise from the rostral medial accessory olive (MAO) and the ventral lamella of the principal olive (PO) while projections from the lobule VII arise from the caudal MAO (Apps and Hawkes, 2009).

### Tactile signals from whiskers

Both anatomical and electrophysiological studies indicate that the cerebellum receives tactile whisker information and is involved in its processing. In particular, stimulation of the whiskers induces simple and/or complex spikes electrophysiological activity in Crus I and Crus II (see review by Bosman et al., 2010, 2011).

Sensory inputs from the whiskers enter the trigeminal nuclei (TGN) (Stuttgen et al., 2008; Schroeder and Hartmann, 2012) and reach the cerebellar cortex via different pathways. Mossy fibers from the trigeminal nuclei project to lobule VII, as well as Crus I, Crus II, lobules IX, X, and to a lesser extent to the flocculus and paraflocculus (Yatim et al., 1996). The trigeminal nuclei also projects to the superior colliculus, which sends afferents to the pontine nuclei (NRTP) and the inferior olive (see Figure 3 and Bosman et al., 2010). The basilar pontine nuclei (BPN) receive whisker inputs both from direct TGN projections, and from the whisker sensory and motor cortices (S1 and S2, M1). The BPN also receive projections from other structures conveying whisker-related information such as motor and sensory whisker cortices as well as the superior colliculus, (Burne et al., 1981; Diamond et al., 2008), which receives inputs from TGN (May, 2006).

Concerning the climbing fiber projections, the three main nuclei of the inferior olive (i.e., MAO, DAO, and PO) receive inputs from TGN (Yatim et al., 1996). Tracing (Swenson et al., 1989) and electrophysiological studies in rats show that they also receive inputs from the whisker sensory cortex (Brown and Bower, 2002).

### Proprioceptive signal

Cerebellum plays a crucial role in proprioception (Bhanpuri et al., 2013). Proprioceptive information from the limbs are conveyed through spino-cerebellar pathways which are not going to be described here except for neck and shoulder proprioception that can be integrated with visuo-vestibular signals to account for possible head vs. body movement during navigation (Gdowski and McCrea, 2000; Bove et al., 2004; Brooks and Cullen, 2009). Proprioceptive information is conveyed by both mossy and climbing fibers (Murphy et al., 1973; Swenson and Castro, 1983). Mossy fiber information from neck and shoulder is relayed in the ECuN, a subnucleus of the dorsal column nuclei (DoCN) and projects mainly to lobule IX (Quy et al., 2011), lobule VII, Crus I, Crus II, paraflocculus and paramedian lobule (Huang et al., 2013). It has been shown that climbing fibers conveying this proprioceptive information reach the same zones as those innervated by mossy fiber at least in the anterior cerebellum (Murphy et al., 1973).

### Multimodal information integration

The description above reveals that multiple sources of self-motion information converge at different anatomical locations en-route to the cerebellum.

*The superior colliculus* has been described as a multi-sensory integrator (see for example Meredith and Stein, 1983, 1986; review in DeAngelis and Angelaki, 2012). It receives visual inputs from the retina and cortices. It is likely to receive visuo-vestibular information through its connection with PrH (Figure 2). It also receives whisker signals through the TGN (Figure 2).

#### The prepositus hypoglossi nucleus (PrH)

Besides the superior colliculus, the PrH appears to be an important precerebellar nucleus that sends multimodal information to the cerebellum (McCrea and Horn, 2006). It receives inputs from both vestibular nuclei and AOS. The efferent connections of the AOS not only convey visual-oculomotor signal but also contribute to visuo-vestibular interaction (Giolli et al., 2006).

It has extensive projection to the uvula-nodulus, the flocculus and paraflocculus, the oculomotor cerebellum (Lobule VII) and Crus I (Barmack, 2003; Ruigrok, 2003; McCrea and Horn, 2006; Voogd and Barmack, 2006) as well as Crus II in primates (Belknap and McCrea, 1988). Therefore, the PrH belongs to a network involved in visual, oculomotor, vestibular, and proprioceptive information integration.

#### The cerebellar cortex

As the caudal medial accessory olive receives afferents from the superior colliculus (receiving retinal and tactile signals) and the dorsal column nuclei (neck proprioception), climbing fiber inputs to lobule VII are modulated by multi-source signals (Azizi, 2007). Interestingly, whisker inputs also reach lobule VII (Bower and Kassel, 1990). Whisking movements are closely coordinated with head movements and such coordination is essential during navigation. The integration of whisker and head movements could be mediated by lobule VII (Hartmann, 2011).

Lobule IX and X receive vestibular inputs (Barmack, 2003) and proprioceptive neck signals related to body-head-position via the external cuneate nucleus which is part of the Dorsal Column Nuclei (Quy et al., 2011).

Purkinje cells of the cerebellar cortex receive convergent inputs of the same multi-sensorial information both from mossy and climbing fiber inputs. Brown and Bower described a convergence of mossy fibers and climbing fibers at the level of the Purkinje cells in the lateral hemispheres of the rats (Crus IIa) after peripherical tactile stimulation (Brown and Bower, 2001). This multisensory information is also conveyed directly from collaterals of these two inputs to the cerebellar nuclei (Sugihara et al., 1999).

#### Integration of the sensory and efference copy signals

When a motor command is sent to an effector, a copy of that command called “efference copy” is sent to the cerebellar cortex via the pontine nuclei (Angel, 1976; Miall and Wolpert, 1996). It is classically proposed that the cerebellar processing of the efference copy provides an expected sensory outcome or “corollary discharge,” which can be compared to the actual sensory consequences of the motor command (Miall and Wolpert, 1996; Blakemore et al., 2001; see review by Stock et al., 2013). Recently Huang et al. (2013) reported that the same granule cells receive both upper body proprioceptive information from ECuN and cortical afferents from an area associated with upper body motor control via the BPN. This convergence is observed in several cerebellar lobules, especially in paramedian lobule, paraflocculus and Crus II. By showing that granule cells of those lobules receive in parallel efference copy and sensory information originating from the same part of the body, those results provide a neural basis for the integration of the two types of information. Besides results in rabbits suggest that efference copy and sensory signal could also terminate on the same Purkinje cells. Indeed, Winkelman and Frens (2006) showed that climbing fibers reaching the flocculus, and previously reported as encoding the retinal slip only, also receive an oculomotor component. Those data thus suggest two levels of convergence of the sensory and motor efference inputs at the granule cells on one hand and via the climbing fiber pathway on the other hand.

In conclusion, the cerebellum appears to be in a position to combine and weight multi-sensory signals originating from various sources. Interestingly, this multisource signal is conveyed redundantly by the MF and the CF inputs. In the following sections, we will question how such multi-source self-motion information arriving in the cerebellum might be processed and then conveyed to spatially modulated cells well described in navigation circuits.

## What computations are performed in the cerebellum during self-motion?

To build a unified representation of the body in space, the brain needs to compare and integrate signals coming from different sensors and from the motor cortex (the efference copy). However, information coming from each modality is intrinsically ambiguous: first, it is generated by sensors located in different parts of the body (e.g., head, neck, limbs), it is therefore measured in relation with the organ and does not give direct access to whole body motion in space; second, each sensory signal can be insufficient to distinguish self-motion from external information by itself (e.g., linear acceleration vs. gravity in the vestibular information; optic flow generated by self-motion vs. the motion of a large object in the environment). Such ambiguities may only be resolved by combining several signals arising from different sensory modalities. The convergence of multi-source signals in the cerebellum described above is likely to contribute to this disambiguation, and provide the navigation structures with reliable self-motion information. That is what we describe in the following.

### Reference frame conversions

Locating oneself is only meaningful relative to a spatial reference frame, i.e., a coordinate system into which spatial information is coded. It is defined by the origin on which it is centered, i.e., the point in space relative to which positions are measured, and by a set of axes corresponding to the three directions of space. A reference frame can be centered on the subject (egocentric reference frame) and more precisely on different parts of the subject (e.g., head vs. trunc-centered reference frame) or on the external world (allocentric reference frame).

Sensory information generated by sensors located in different parts of the body (e.g., head, neck, limbs) are initially encoded in the respective reference frames of its sensors. For example, as vestibular organs are located in the head, vestibular signal is detected in head coordinates. To allow the combination and/or comparison of different sensory signals, those must be expressed in a common reference frame. For example, to compute the movement of the whole body in space, vestibular information needs to be integrated relative to the body (taking into account the relative position of the head and the body given by the neck curvature) and also to the world (taking into account gravity).

Converting the signal initially in head-fixed coordinates into a signal in body-frame and world-frame coordinates are not necessarily successive computations. Several recent studies showed that these two reference frame transformations may occur (in parallel) in different cerebellar subregions (see review in Rochefort et al., 2013). Indeed, signals related to head-to-body frame transformation have been detected in the cerebellar fastigial nucleus (Kleine et al., 2004; Shaikh et al., 2004; Brooks and Cullen, 2009), and Purkinje cell activity has been shown to be modulated by head-to-body position, a signal required for such a transformation, in the cerebellar anterior lobules IV and V of decerebrate cats (Manzoni et al., 1999). On the other hand, the head-to-world reference frame conversion has been proposed to occur in the lobules IX and X of the cerebellar cortex (Yakusheva et al., 2007; Angelaki et al., 2010). We have already proposed that, once adequately transformed, the vestibular information fuels the neuronal circuits of navigation, in particular the hippocampus (Rochefort et al., 2013).

### The cerebellum as an adaptive filter

In parallel with functional and experimental descriptions seen above, computational models of cerebellar function have also been proposed (Marr, 1969; Albus, 1971; Ito, 2002). Recent descriptions of the cerebellar micro-circuit features (Cerminara et al., 2013) led to the proposal of an updated and more generalistic model of the cerebellar function as an adaptive filter (Dean and Porrill, 2010).

A filter transforms an input signal into an output signal. In the context of the cerebellar microcircuits, the input signal comes from the mossy fibers (MF) and is distributed onto different granule cells (GC), each one extracting a component of this signal. Interestingly, a GC combines the signal from up to four MF in average (Albus, 1971) and therefore not only analyzes the input signal but also combines several ones (see Section Integration of the sensory and efference copy signals in this paper). Those components of the signal are transmitted through parallel fibers (PF) to dendrites of Purkinje cells (PC) via PF-PC synapses. One Purkinje cell therefore receives the signal from different parallel fibers which are weighted at the PF-PC synapse and recombined to form the filter output (PC simple spike).

The filter is adaptive because the weight at the PF-PC synapses, corresponding to the synapse efficiency, can be modified through bi-directional plasticity (LTP and LTD). In Dean and Porrill's model, these plasticities are under the control of a teaching or error signal coming from the climbing fibers input. The weight adjustment follows the covariance rule: a PF signal that is positively correlated with an error signal has its weight reduced (through LTD), whereas a signal that is negatively correlated with an error signal has its weight increased (through LTP). The climbing fiber signal is thus considered to implement a supervised learning. Interestingly, LTP at the PF-PC synapses can be induced by PF activity alone (Belmeguenai and Hansel, 2005). In this condition, the increase of the filter weights would not be exclusively under the control of the climbing fibers, and could occur through monosynaptic plasticity/LTP at the PF-PC synapse, which could implement a non-supervised learning. It is still unclear which specific role each type of learning could play and how these two could combine in the same model. The extensive literature on the manipulation of cerebellar LTP and/or LTD in genetically modified mice (Gao et al., 2012) could help to tackle this question.

### Sensorimotor prediction

With the present model, Dean and Porrill propose possible neural implementations of forward model architectures for taking into account self-induced signals and detecting novelty in particular in the rat whisker system (Anderson et al., 2012; Porrill et al., 2013). This model accounts for the known competence of the cerebellum in sensorimotor prediction (Blakemore et al., 2001) and fits with the recent findings that the primate cerebellum encodes unexpected self-motion (Brooks and Cullen, 2013). Recording from monkeys during voluntary and externally applied self-motion, Brooks and Cullen (2013) demonstrated that the cerebellum can distinguish unexpected self-motion resulting from external factors and self-motion generated by voluntary actions by making predictions about the expected sensory state. Cullen et al. (2011) propose the possible production of a cancelation signal to suppress self-generated vestibular stimulation due to active movements. This computation implies using the efferent copy of the motor command to model a sensory prediction (expected sensory feedback) then comparing this expected feedback to the actual sensory signal (Roy and Cullen, 2004). This cerebellar internal model could be responsible for the error prediction necessary to finely tune motor movements, as well as to perceive oneself in space correctly.

In line with this hypothesis, Bhanpuri et al. (2013) and Bhanpuri et al. (2012) showed that cerebellar patients have proprioceptive deficits compared with controls during active movement, but not when the arm is moved passively. They also find that similar deficits can be reproduced in healthy subjects by making them moving in a force field with unpredictable dynamics. The authors propose that it is the predictability of self-generated movement rather than muscle activity alone which is important to enhance proprioception, and conclude that the proprioceptive deficit of cerebellar patients in active conditions is consistent with disrupted movement prediction. Christensen et al. (2014) also found that cerebellar patients had no beneficial influence of action execution on perception compared with healthy controls. Cerebellum is thus proposed to be crucial to take into account self-generated movement to enhance somato-sensory perception related to those movements.

The adaptive filter not only accounts for those results but also extend their conclusions from the mere cancelation of the autogenerated signal to the possible detection of an external novelty signal. Here, we discuss the possible generalization of this model to the detection of new features of the external environment in the context of navigation, whatever the sensory modality.

### Novelty detection

Whether we consider vestibular, acoustic, visual or whisker tactile signal, we can consider sensory information received by the cerebellum as two-dimensional: that which is internally generated by voluntary self-motion and that which is externally generated (for instance passive self-motion due to unexpected external events). In externally generated self-motion, we can further dissociate sensory inputs which already occurred and are thus predictable from newly occurring features (novelty). The sensory prediction performed by the cerebellum takes into account both the motor efference and the current sensory state of the navigator. With this double input it is possible to predict the future sensory inputs due to internally generated self-motion by “adding” to the current sensory state the evolution of this current state expected from the intended movement (see Miall and Wolpert, 1996). It might also be possible to predict the expected future sensory state due to the navigation context already encountered (Anderson et al., 2012). In a non-navigational context, the cerebellum (lobule VII Crus I) was found engaged in predicting the position change over time of an occluded target based on its visual speed before occlusion (O'Reilly et al., 2008). The comparison between the actual sensory state and the predicted sensory signal will then provide the novelty information. Accordingly, Naatanen and Michie (1979) proposed that the cerebellum detects “discordances between the input from the deviant event and the sensory memory representation of the regular aspects of the preceding stimulation.” Thus, cerebellar patients were impaired in the cortical processing of deviant somatosensory inputs presented in a regular context (Restuccia et al., 2007), suggesting that the cerebellum could be the site where novelty is extracted by comparing actual stimuli with predictable ones.

**Figure 4:**
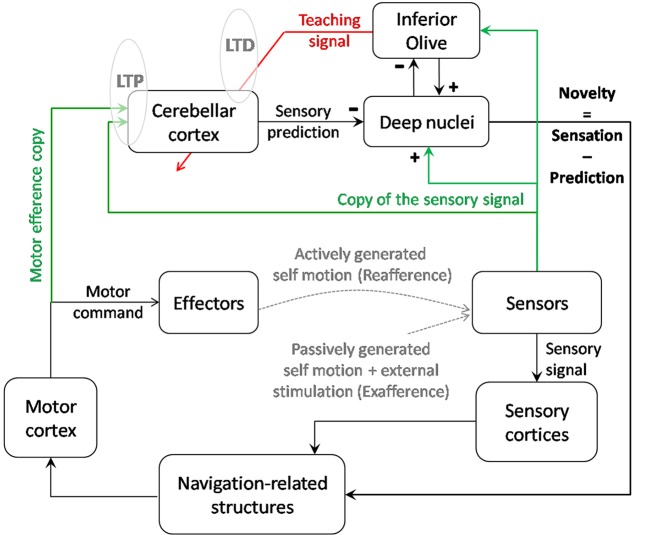
**Contribution of cerebellar computation to navigation**. Cerebellar cortex as an adaptive filter could transform an input signal consisting of self-motion sensory signals and a motor efference copy into a prediction of the sensory signal expected from voluntary movements and the previous sensory state. By comparing the sensory prediction with the actual sensory signal, the cerebellar output helps the system to detect novelty in the environment. Thanks to a parallel output sent to the inferior olive, this novelty signal is modified into a teaching signal which, if repeated in correlation with the sensory inputs, could contribute to modify the cerebellar model of prediction through LTD. Reciprocally, repeated inputs to the cerebellum may trigger LTP at the synapses gating those inputs and act as an unsupervised learning of the most relevant inputs. Depending on the type of signal (vestibular, tactile…) and possibly on the targeted lobules (IX-X vs. VII, crus I, and II), the output of the cerebellar computation could cancel the signal induced by voluntary self-motion and allow detection of novelty. This computation could have a double role: stabilizing perception during voluntary navigation and informing the navigator about the necessity to update his/her relative position in the context.

This comparison has been proposed to be performed by the superior colliculus (Porrill et al., 2013). We propose this could also occur in the deep cerebellar nuclei as those receive adequate projections to perform this comparison: on one hand from Purkinje cells conveying sensory prediction to inhibitory synapses; on the other hand, from mossy fibers conveying sensory inputs to excitatory synapses. Interestingly, the inferior olive could also have a role of comparator since it receives on one hand inhibitory inputs from the deep cerebellar nuclei (Angaut and Sotelo, 1989), which could convey sensory prediction, and on the other hand actual sensory signals (see Figure 4).

The new sensory inputs containing (1) actively generated, (2) passively generated, and (3) external information, novelty can arise from any of those three sources, e.g., (1) internal modification of the muscle strength, (2) obstacle modifying/blocking unexpectedly the trajectory of a limb, (3) new object in the navigator environment inducing tactile stimulation and therefore influence space modulated cells.

## From cerebellum to spatial knowledge

We saw previously that, once adequately transformed in the cerebellum, the sensory information may provide the neuronal circuits of navigation, in particular the hippocampus, with reliable self-motion information or novelty information. We now describe how this information may influence navigation-related cells.

To our knowledge, only one study has reported the consequence of cerebellar impairment on the activity of navigation-related cells (Rochefort et al., 2011). In this study, hippocampal place cells (review by O'Keefe, 1979) were recorded in freely exploring L7-PKCI mice, which lack PKC dependent LTD at the parallel fiber—purkinje cell synapses (De Zeeuw et al., 1998). The results revealed an implication of cerebellar LTD in maintaining the hippocampal spatial map when the mice had to rely on self-motion information. This finding first raised the question of how such self-motion information, processed by the cerebellum, may influence place field properties.

### Influence of self-motion information on place cell firing

The role of self-motion information in the control of place fields has originally been demonstrated from the observation that place fields were maintained in the dark if the animal stayed in the arena when the light was switched off (Quirk et al., 1990). If the animal was placed in the arena directly in the dark, i.e., in the absence of any visual information, the place field appeared at a random location. This suggested that self-motion information was used to maintain the location specific firing of place cells previously recorded in the light (Quirk et al., 1990). Among the different self-motion inputs, vestibular information was shown to be important for hippocampal spatial representation since a temporary inactivation of the vestibular system by tympanic injection of tetrodotoxin (TTX) dramatically altered the activity pattern of place cells (Stackman et al., 2002; see review in Smith and Zheng, 2013).

Recently, the development of recording techniques for head-fixed mice navigating in virtual environments has given the opportunity to further dissect the contribution of non-vestibular self-motion signals to place cell firing (Chen et al., 2013; Ravassard et al., 2013). In such an experimental setup, head-fixed mice are trained to run on an air-cushioned ball surrounded by a screen showing a first-person perspective view of a virtual linear track or maze. The movement of the viewpoint corresponds to the movement of the ball, and the mouse receives visual and other non-vestibular self-motion cues such as proprioceptive and efference copy inputs. Despite the absence of vestibular motion signals, normal place cell firing was found. Amongst these place cells, visual information alone was sufficient to sustain location-specific firing in 25% of place cells and additional movement-related information was required for normally localized firing by the remaining 75% of place cells (Chen et al., 2013). Comparing the hippocampus spatiotemporal selectivity in virtual reality and similar real world navigation tasks, Ravassard et al., reported that distal visual and non-vestibular self-motion cues are sufficient to generate a cognitive map but that vestibular and other sensory cues present in the real world, such as tactile and olfactory cues, are necessary to fully activate the place cell population (Ravassard et al., 2013).

The importance of tactile whisker signals in navigation processes has also been described. Hippocampal CA1 neurons have been shown to encode tactile stimuli in conjunction with the location in which they appeared (Itskov et al., 2011). Diamond et al. showed that a representation of the surrounding world is built through a whisker-mediated sense of touch (Diamond et al., 2008).

**Figure 5:**
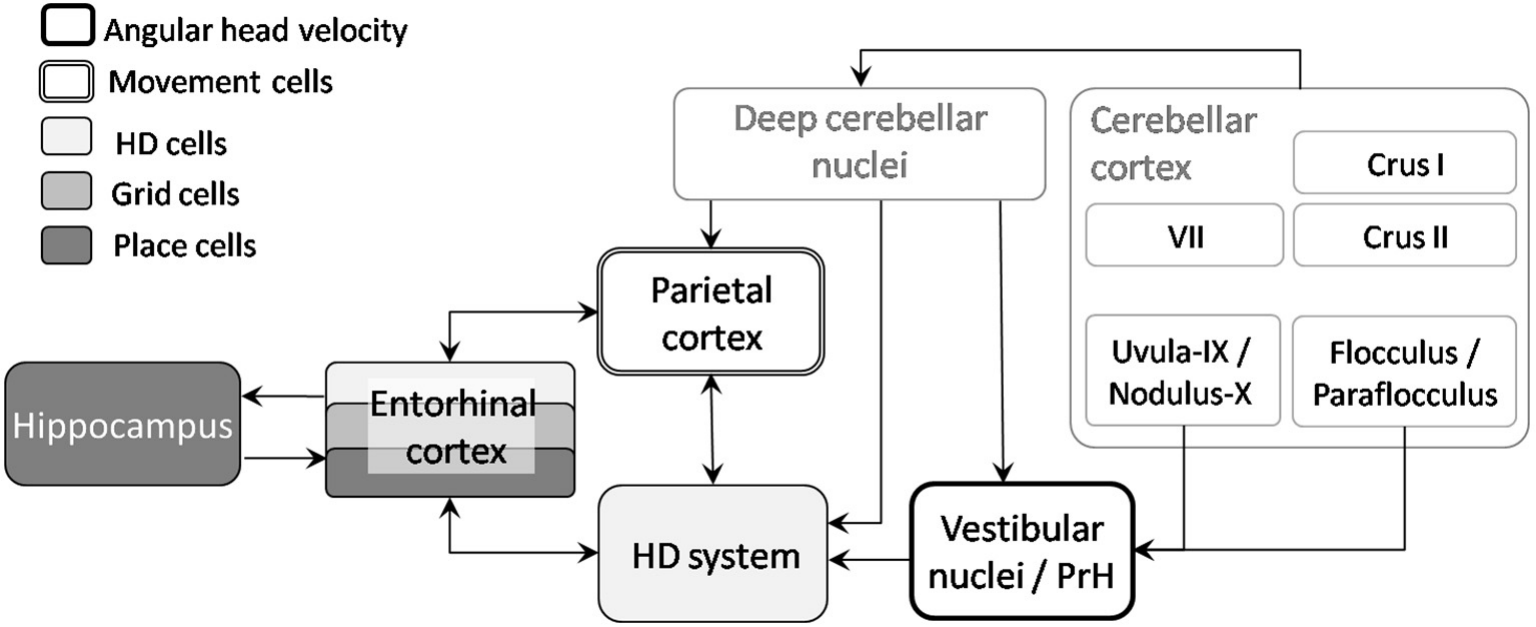
**From cerebellum to navigation-related structures**. Two pathways could sustain the influence of the cerebellum on place cells activity: the projection of lobule IX-X-floculus-parafloculus through vestibular nuclei to the head direction cells system and projection of other cerebellar lobules via deep cerebellar nuclei to the parietal cortex and HD system. AHV, Angular head velocity; HD, Head direction; PrH, Prepositus Hypoglossi Nucleus.

Therefore, self-motion signals can clearly influence space hippocampal coding. Nevertheless, as no direct pathway has been anatomically described between the cerebellum and the hippocampus (see review in Rochefort et al., 2013), we propose two potential pathways that could provide a neuro-anatomical substrate allowing for cerebellar interactions with navigation-related cells (see Figure 5):

- The projection of the lobule IX-X-floculus and parafloculus to vestibular nuclei and PrH which directly feed the head-direction (HD) cells system (Shinder and Taube, 2010);
- The projection of posterior cerebellar lobules (including VII, Crus I, and Crus II), through the deep cerebellar nuclei and ventro and centro-lateral thalamus (Giannetti and Molinari, 2002), to the parietal cortex which contains “movement cells” (Whitlock et al., 2012) as well as “path cells” (Nitz, 2006, 2012).

### Influence of self-motion information on HD firing

*Head direction* (HD) cells fire when a rat's head is facing a specific direction relative to the environment, irrespective of its location or whether it is moving or still (Taube et al., 1990a,b). These cells were first discovered in the dorsal portion of the rat presubiculum, often referred to as the post subiculum (PoS) (Ranck, 1984), but have since been found in multiple structures which are anatomically interconnected: anterior dorsal thalamic nucleus (Taube, 1995), lateral mammillary nuclei (Stackman and Taube, 1998), lateral dorsal thalamus (Mizumori and Williams, 1993), retrosplenial cortex (Chen et al., 1994; Cho and Sharp, 2001), entorhinal cortex (Sargolini et al., 2006) and even striatum (6% of HD) (Mizumori and Williams, 1993; Wiener, 1993). Lesion and electrophysiological studies have shown that the head-direction signal travels from the dorsal tegmental nucleus, to the hippocampus, through the hypothalamus (mammillary nucleus), the antero-dorsal thalamus and the retrosplenial, subicular and entorhinal cortices (Taube, 2007). The dorsal tegmental nucleus receives indirect inputs from vestibular nuclei via the supragenual nucleus (Clark et al., 2012) and the Nucleus prepositus hypoglossi (McCrea and Horn, 2006; Clark et al., 2012). It is important to note that most of the connections between these structures are bidirectional and that the transfer of head direction information is not necessarily unidirectional.

Despite the strong reliance of the HD system on landmark cues (Goodridge and Taube, 1995), removing all the visual cues or turning off the light does not strongly affect HD cell firing in the post subiculum and thalamus (Taube et al., 1990b; Mizumori and Williams, 1993; Goodridge et al., 1998). Using Fischer albino rats, Knierim et al. (1998) found that self-motion inputs could predominate over the visual landmarks when a conflict was created between both types of information and the mismatch was larger than 45°. This suggests that self-motion information can maintain HD signals to some extent in the absence of reliable visual information. Numerous rodent studies have demonstrated that vestibular signals influence landmark navigation (see review Yoder and Taube, 2014). The PrH is one of the two main nuclei that provide multimodal information to the HD cell circuit through connections with the dorsal tegmental nucleus, which is considered as a putative location of head direction signal generation (Yoder and Taube, 2014). Consistently, vestibular lesions abolished the directional firing properties of HD cells, demonstrating that the HD signal critically depends on vestibular information (Stackman and Taube, 1997; Stackman and Herbert, 2002). The importance of proprioceptive (and motor command) information was shown by recording HD cells in the antero-dorsal thalamus in two environments connected by a passageway. In the dark, if the animal actively walked from one environment to the other, HD cells could partly retain their preferred direction between the two environments. However, this was no longer the case if animals were passively transported in the dark from one environment to another, conditions in which only the available information was the vestibular signals (Yoder et al., 2011). This showed the requirement to combine different types of self-motion information (vestibular and proprioceptive) to maintain HD signals in the absence of visual information. Head direction cell were recently shown to be sensitive to optic flow information as well. Rats were freely moving in an arena where the repetitive background (not usable as a landmark) of the cylinder wall was slowly rotated, thus providing a continuously drifting optic flow. Recordings in the antero-dorsal thalamus showed that HD cells exhibit a significant drift in the same direction as the rotating background (Arleo et al., 2013).

### Motion-related cells in the parietal cortex

Interestingly, in the parietal cortex which constitutes the second possible pathway from the cerebellum, at least two types of cells have recently been discovered to be modulated by the displacement of the animal. The first type, which we will call “movement cells” fires whenever the animal moves in a specific direction, irrespective of its location and heading, for example forward or rightward (McNaughton et al., 1994; Whitlock et al., 2012). These cells fire independently from context since their activity pattern is preserved in different environments and seems to depend on self-motion information. When the animals perform a specific sequence of movements (in a hairpin maze), these cells can acquire new movement specificity (from leftward to backward for example). This last finding led the authors to propose that the activity of these cells is determined by the organization of actions. The second type, which we will call “path cells,” has been identified by Nitz (2006) as encoding the state of progression through a route. Recording parietal neurons while rats traversed squared spiral tracks, he further dissected this finding by showing that those neurons simultaneously encoded the rat's position in several coexistent reference frames: linear segments, square loops and full route (Nitz, 2012). Such encoding may contribute to relate different parts of a route by taking into account the motion of the animal in the maze.

### Self-motion information influence on grid cell firing

Finally, the entorhinal cortex receives convergent inputs from both the parietal cortex and the retrosplenial cortex, the latter being part of the HD system (Taube, 2007). Grid cells were first discovered in the dorsal medial entorhinal cortex (Fyhn et al., 2004; Hafting et al., 2005), but have also been found in the pre- and parasubiculum (Boccara et al., 2010). Each grid cell fires in several locations in an environment, with the locations forming a regular pattern as though they were nodes on a triangular grid (Fyhn et al., 2004; Hafting et al., 2005). They are most abundant in layers II and III of the medial entorhinal cortex (Sargolini et al., 2006), which receive convergent inputs from the retrosplenial and the parietal cortices and sends major projections to the hippocampus, but are also found in the layers V/VI, which receive inputs from the hippocampus.

Several observations indicate that grid cell firing depends primarily on self-motion information (Hafting et al., 2005). First, a grid cell fires in all environments (in contrast to the hippocampus where an environment is encoded by a subset of active cells), and the spacing of a grid cell is independent of the context. Second, grid fields appear relatively independent of specific landmarks since they can be observed immediately as an animal starts to explore an environment, and the grid pattern does not change drastically in the dark. Grid cell are thus proposed to encode a metric system for spatial navigation, whereby the animal can update its own location using self-motion information (path integration) (Jeffery and Burgess, 2006; Moser and Moser, 2008). This is also consistent with the finding that entorhinal cortex lesions alter self-motion based navigation (Parron and Save, 2004).

In conclusion, different types of navigation-related cells display modifications of firing in response to the manipulation or suppression of different modalities of self-motion cues. The direct anatomical projections of the cerebellum toward the HD system and the parietal cortex make HD cells and movement cells likely candidates for potential influence of the cerebellum on the hippocampal spatial representation. Studies manipulating sensory signals from different modalities reveal the diversity of information that is taken into account to build mental maps. They also illustrate the necessity to process multi-source information to extract the signal appropriate to shape the firing characteristics of the spatially modulated cells. We speculate that what will arise in the navigation structures from the cerebellum will convey the novelty signal necessary to implement an update over time of position in the context of navigation and allow stabilization of perception during voluntary navigation.

### Conflict of interest statement

The authors declare that the research was conducted in the absence of any commercial or financial relationships that could be construed as a potential conflict of interest.
